# Fatty liver index correlates with non-alcoholic fatty liver disease, but not with newly diagnosed coronary artery atherosclerotic disease in Chinese patients

**DOI:** 10.1186/1471-230X-13-110

**Published:** 2013-07-08

**Authors:** Zhao-Yan Jiang, Chen-Ying Xu, Xian-Xing Chang, Wei-Wei Li, Lu-Ying Sun, Xiao-Bo Yang, Li-Fen Yu

**Affiliations:** 1 Department of Surgery, Shanghai Institute of Digestive Surgery, Shanghai, 200025, China; 2 Department of Gastroenterology, Ruijin Hospital, Shanghai Jiao Tong University School of Medicine, Shanghai, 200025, China

**Keywords:** Fatty liver index, Non-alcoholic fatty liver disease, Coronary artery atherosclerotic disease, Coronary angiography

## Abstract

**Background:**

Fatty liver index (FLI) was recently established to predict non-alcoholic fatty liver disease (NAFLD) in general population, which is known to be associated with coronary artery atherosclerotic disease (CAD).

This study aims to investigate whether FLI correlates with NAFLD and with newly diagnosed CAD in a special Chinese population who underwent coronary angiography.

**Methods:**

Patients with CAD (n = 231) and without CAD (n = 482) as confirmed by coronary angiography were included. Among them, 574 patients underwent B-ultrosonography were divided into NAFLD group (n = 209) and non-NAFLD group (n = 365). Correlation between FLI and NAFLD was analyzed using pearson’s correlation. The associations between FLI and NAFLD as well as CAD were assessed using logistic regression. The predictive accuracy of FLI for NAFLD was evaluated using receiver operating characteristics (ROC) curve analysis.

**Results:**

FLI was significantly higher in NAFLD group (37.10 ± 1.95) than in non-NAFLD group (17.70 ± 1.04), P < 0.01. FLI correlated with NAFLD (r = 0.372, P < 0.001). The algorithm for FLI had a ROC-AUC of 0.721 (95% CI: 0.678–0.764) in the prediction of NAFLD. Logistic regression analysis showed that FLI was associated with NAFLD (adjusted OR = 1.038, 95% CI: 1.029-1.047, P < 0.01). The proportion of patients with CAD did not differ among the groups of FLI ≤ 30 (32.3%), 30-60 (31.0%), and ≥60 (35.3%). No significant association was found between FLI and CAD (adjusted OR = 0.992, 95% CI: 0.981-1.003 in men and OR = 0.987, 95% CI: 0.963-1.012 in women, P > 0.05).

**Conclusions:**

FLI showed good correlation with NAFLD in patients who underwent coronary angiography, but not with newly diagnosed CAD. This might be underestimated because some patients in non-CAD group may have other underlying cardiovascular diseases.

## Background

Non-alcoholic fatty liver disease (NAFLD) is a progressive problem in Asia-Pacific region [1]. The prevalence of NAFLD is increasing to about 15-20% in cities like Shanghai, Guangzhou and Hong Kong in China recently [2]. It is associated with metabolic disorders of lipids, hypertension, diabetic mellitus and coronary arterial atherosclerotic disease (CAD). CAD is one of the leading causes of death from NAFLD [3-5]. Thus, prediction of NAFLD at earlier stage is important in prevention of the inherent process of NAFLD and the associated fatal diseases such as CAD.

So far, the diagnosis of NAFLD is based on computerized tomography (CT) scanning, magnetic resonance spectroscopy (MRS), B-ultrasonography and even liver biopsy. However, most subjects with NAFLD do not have symptoms, especially at the very early stage, which limits the early detection and prevention of NAFLD. Bedogni et al [6] established a formula to calculate fatty liver index (FLI) based on triglycerides, body mass index (BMI), gamma-glutamyltrasnferase (GGT) and waist circumference (WC) to predict NAFLD in Italian population. These variables are risk factors for CAD as well. Therefore, whether FLI can be used as a marker for the prediction of CAD development is worth being explored.

The aim of this study is to evaluate whether FLI is able to predict NAFLD in a special Chinese population who underwent coronary angiography and to investigate whether FLI is associated with newly diagnosed CAD.

## Methods

### Study subjects and procedure

This was a cross-sectional study. From January 2007 to September 2011, consecutive patients with suspected CAD (ie, those with angina or abnormal exercise stress test results) who presented for the first time for coronary angiography were recruited in this study. Patients underwent coronary angiography for reasons as congenital heart disease, congestive heart failure, cardiomyopathy and valvular heart diseases were excluded. According to criteria of National Institutes of Health Clinical Research Network [1,7], patients with alcohol intake >140 g/week in male or >70 g/week in female were not included in the study. Patient with other causes such as viral hepatitis, drug induced hepatitis, autoimmune hepatitis, etc., were not included either. Finally, 713 patients were included in the study. In accordance with American College of Cardiology/American Heart Association guidelines, patients were defined as CAD positive (CAD group: n = 231, male/female: 171/60) if equal to or more than 50% diameter stenosis in any one of the major coronary arteries was found on coronary angiography. If no stenosis was observed, those patients were defined as non-CAD (non-CAD group: n = 482, male/female: 215/267). We did not include patients with stenosis less than 50%, because these lesions may progress, and eventually, patients with no significant obstructions have significantly more cardiovascular events during follow-up than those with truly normal coronary angiograms [8].

Among 713 patients, 574 patients underwent B-type ultrasonography, who were accordingly classified into NAFLD group (n = 209, male/female: 113/96) and non-NAFLD group (n = 365, male/female: 193/172). The diagnostic criteria for steatosis under B-type ultrasound was indicated by diffusely increased echogenicity (“bright”) liver with liver echogenicity greater than kidney, with vascular blurring, and deep attenuation of ultrasound signal [9,10]. BMI was calculated by weight/height^2^ (kg/m^2^). FLI was calculated by previous reported formula [6].

The study protocol was approved by Ethical Committee at Ruijin Hospital and written informed consents to participate in the study were obtained from all the patients. The study was performed in accordance with the Declaration of Helsinki.

### Statistical analysis

Continuous variables were presented as means ± S.E.M and compared by t-test between groups. Categorical variables were summarized as numbers of subjects (percentage) and compared by *χ*^2^ test between groups. Cochran-Armitage trend tests were also performed if there were more than two categorical variables. The predictive accuracy of FLI for NAFLD was evaluated using receiver operating characteristics (ROC) curve analysis by DeLong method. In addition, an area under the ROC curve (AUC) with 95% confidence intervals (CI) was calculated for different markers. A correlation between FLI with NAFLD was analyzed with Pearson’s correlation. Associations between FLI and NAFLD as well as CAD were analyzed by logistic regression analysis, respectively. Covariates included age, gender, FLI, ALT, AST, alkaline phosphatase (ALP), total cholesterol (TC), HDL-C, LDL-C, fasting plasma glucose (FPG), history of hypertension, history of diabetic mellitus, and history of dyslipidemia. Data were expressed as odds ratio (OR) with 95% CI were calculated. All analyses were carried out with SPSS version 17.0. The statistical significance was set at *P* < 0.05. All P values were two-sided.

## Results

### The association between FLI and NAFLD

**Figure 1:**
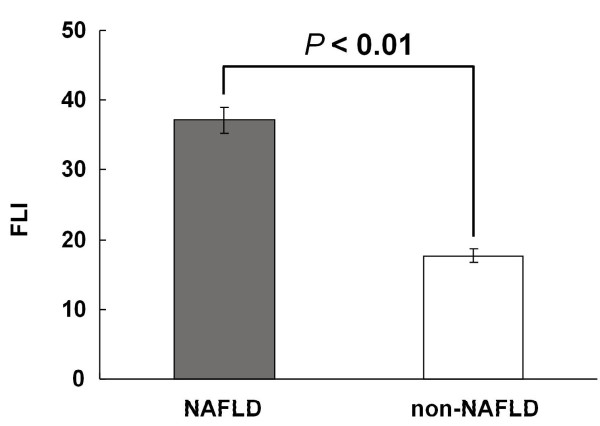
**Fatty liver index (FLI) was significantly higher in patients with NAFLD than in patients without NAFLD (non-NAFLD), 37.10 ± 1.95 *vs. *17.70 ± 1.04, *P*** **< 0.01.**

The clinical characteristics between NAFLD and non-NAFLD groups were shown in Additional file 1: Table S1. Patients with NAFLD had significantly higher FLI (37.10 ± 1.95) than patients without NAFLD (17.70 ± 1.04), *P* < 0.01, Figure 1. According to Bedogni’s ‘rule in and rule out’ criteria [6], we divided the patients into three categories: FLI ≥ 60, 30 < FLI < 60, and FLI ≤ 30. The clinical characteristics among three FLI categories were shown in Additional file 1: Table S2. The proportion of patients with NAFLD was 74.6% (53/71) in group with FLI ≥ 60, 48.3% (56/116) in group with FLI 30-60, and 25.8% (100/387) in group with FLI ≤ 30 (Cochran-Armitage trend test: *P* < 0.01), Table 1.

**Table 1:** Distribution of NAFLD and non-NAFLD subjects in different FLI categories

	**FLI ≤ 30**	**FLI 30-60**	**FLI ≥ 60**	*P *value*
	**(n = 387)**	**(n = 116)**	**(n = 71)**	
NAFLD	100 (25.8%)	56 (48.3%)	53 (74.6%)	<0.01
Non-NAFLD	287 (74.2%)	60 (51.7%)	18 (25.4%)	<0.01

*Cochran-Armitage test for trend between three FLI categories.

**Figure 2:**
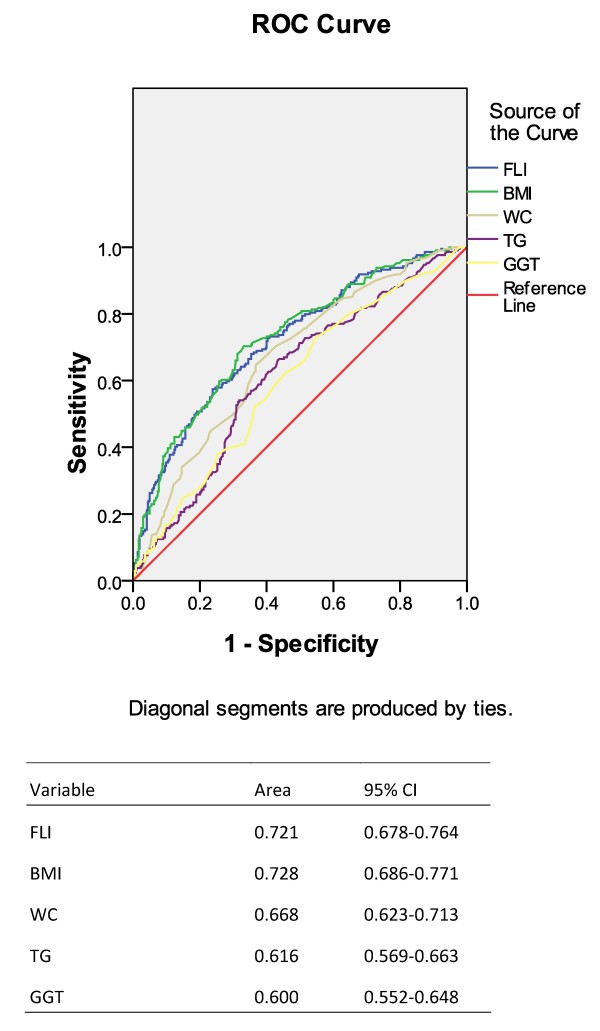
**AUC-ROC of FLI for prediction of fatty liver (value = 0.721, 95% CI: 0.678-0.764).** FLI, fatty liver index; AUC, area under curve; ROC: receiver operating curve.

The model for FLI had a ROC-AUC value of 0.721 (95% CI: 0.678-0.764, Figure 2) in the prediction of NAFLD. The accuracy of FLI in the prediction of NAFLD was better than any single variable in the formula except BMI (Figure 2). Pearson’s correlation showed FLI correlated with NAFLD (r = 0.372, *P* < 0.001). Using multivariate Logistic regression analysis, we found that FLI was associated with NAFLD (age-, sex-adjusted OR = 1.038, 95% CI: 1.029-1.047, *P* < 0.01) (Table 2). History of dyslipidemia, diabetic mellitus, and hypertension were also factors associated with an increased risk of NAFLD (Table 2). To minimize the influence of drug therapy on the development of NAFLD, a further analysis adjusted for anti-hyperlipidemia and diabetic mellitus drugs was performed. The result showed that FLI was still associated with NAFLD (adjusted OR = 1.038, 95% CI: 1.028-1.049, P < 0.01).

**Table 2:** Multivariate logistic regression analysis of factors associated with NAFLD

	**OR (95% CI)**	***P *value**
FLI	1.038 (1.029-1.047)	< 0.01
History of hypertension	1.627 (1.051-2.519)	0.029
History of diabetic mellitus	1.890 (1.190-3.003)	0.007
History of dyslipidemia	1.414 (1.006-2.217)	0.047

### Multivariate logistic regression analysis on the factors associated with CAD

The clinical characters between CAD group and non-CAD group were shown in Additional file 1: Table S3. Unexpectedly, no significant difference was observed in the proportion of patients with CAD among groups with FLI ≤ 30, 30-60 and ≥60, which was 32.3% (156/483), 31.0% (45/145) and 35.3% (30/85) (Cochran-Armitage trend test: P > 0.05), respectively (Table 3).

**Table 3:** Distribution of patients with and without CAD in different FLI categories

	**FLI ≤ 30**	**FLI 30-60**	**FLI ≥ 60**	*P *value*
	**(n = 483)**	**(n = 145)**	**(n = 85)**	
CAD (n = 231)	156 (32.3%)	45 (31.0%)	30 (35.3%)	>0.05
Non-CAD (n = 482)	327 (67.7%)	100 (69.0%)	55 (64.7%)	>0.05

*Cochran-Armitage test for trend between three FLI categories.

In the multivariate Logistic regression analysis, we found that both in males and females, older age, higher total cholesterol, were common factors associated with an increased risk of CAD (Table 4). Higher HDL-C was associated with a decreased risk of CAD (Table 4). However, no significant association was found between FLI and CAD in either males or females (adjusted OR = 0.990, 95% CI: 0.980-1.001 in men and OR = 0.988, 95% CI: 0.965-1.012 in women, *P* > 0.05). In this study, there were 85.5% of patients in the non-CAD group and 95.7% of patients in the CAD group prescribed with anti-hyperlipidemia drugs. On the other hand, 12.9% of patients in the non-CAD group and 13.4% of patients in the CAD group were treated with anti-diabetic mellitus drugs (Additional file 1: Table S3). To minimize the influence of drug therapy on the metabolism of liver, a further analysis was performed after adjusted for drug therapy. However, FLI was not associated with CAD in either gender (Table 4).

**Table 4:** Multivariate logistic regression analysis of factors associated with CAD

	**Men (n = 386)**		**Women (n = 327)**	
	**OR (95% CI)**	**OR (95% CI)**^**a**^	**OR (95% CI)**	**OR (95% CI)**^**a**^
Age	1.049 (1.021–1.077)**	1.047 (1.019–1.075)**	1.061 (1.019–1.106)**	1.055 (1.012–1.100)*
Smoking	1.404 (0.760–2.593)	1.390 (0.748–2.582)	0.759 (0.122–4.729)	0.750 (0.120–4.689)
Alcohol intake	0.441 (0.243–0.801)**	0.451 (0.246–0.828)**	3.384 (0.416–27.532)	2.858 (0.357–22.863)
History of hypertension	1.289 (0.714–2.328)*	1.079 (0.579–2.011)	2.774 (0.989–7.779)	1.850 (0.638–5.359)
History of dyslipidemia	2.034 (1.169–3.538)*	1.996 (1.142–3.489)*	1.494 (0.718–3.105)	1.656 (0.781–3.512)
History of DM	0.379 (0.163–0.883)	0.419 (0.146–1.206)	0.612 (0.237–1.581)	0.925 (0.231–3.710)
ALP	1.000 (0.986–1.015)	0.999 (0.984–1.014)	0.997 (0.981–1.014)	0.996 (0.979–1.013)
TC	1.745 (1.393–2.185)**	1.752 (1.395–2.200)**	1.292 (1.007–1.658)*	1.293 (1.005–1.664)*
HDL	0.275 (0.097–0.785)*	0.305 (0.104–0.894)*	0.191 (0.051–0.710)*	0.196 (0.052–0.742)*
FPG	1.282 (1.052–1.563)*	1.264 (1.036–1.543)*	1.224 (0.981–1.526)	1.348 (1.050–1.731)*
FLI	0.990 (0.980–1.001)	0.991 (0.980–1.002)	0.988 (0.965–1.012)	0.985 (0.961–1.010)

a: adjusted for anti-hyerlipidimia and diabetic mellitus drug therapy.

* represents P < 0.05 and ** represents P < 0.01.

## Discussion

Fatty liver is usually diagnosed by CT scanning, MRS or B-ultrosonography. Although pathological diagnosis by liver biopsy is a golden standard, it is invasive and not feasible for large population study. FLI was first established by Bedogni et al [6] based on data from Italian general population. It is a simple way to calculate the possibility of fatty liver with only four parameters and reached an accuracy of 0.84 (95% CI: 0.81-0.87). BMI, WC, TG and GGT are variables usually easy to obtain. However, due to variation of ethnicity, dietary and environmental factors, the cut-off for waist and BMI is different for the Asian people [11,12]. Thus, FLI needs to be validated when used in a different population. With the same diagnostic criteria for fatty liver disease (by ultrasonography) [6], we also showed a good correlation between FLI and non-alcoholic fatty liver disease (pearson’s correlation r = 0.372, P < 0.001) in a special Chinese population who underwent coronary angiography. The accuracy of FLI was relatively lower (AUC-ROC value = 0.721, 95% CI: 0.678-0.764) than that of Bedogni et al’s study, but was nearly the same as that of Korean general population (accuracy of 0.785, 95% CI: 0.728–0.842) [13]. The results of our study provided further evidences that FLI could be considered as an easy and useful marker for screening fatty liver subjects in both general and special population.

Patients with NAFLD were more likely to have CAD as shown in both retrospective and prospective studies [14,15]. CAD was also the common cause of death for NAFLD in population-based cohort studies [16-18] and follow-up studies [19,20]. In a 13-year follow up study [21], CAD was shown to be the main cause of death in patients with NAFLD confirmed by liver biopsy. The underlying mechanism linked between NAFLD and CAD could be insulin resistance, lipid disorders, inflammation, etc [22,23]. Furthermore, NAFLD is considered as the hepatic manifestation of metabolic syndrome [23] which is believed to be a risk factor for CAD. These accumulated evidences suggested that physicians and researchers had gradually drawn attention to the relationship between NAFLD and CAD in recent years.

Based on the good correlation between FLI and NFALD in patients who underwent coronary angiography, the second aim of our study is to investigate whether FLI was related with CAD. In Calori et al’s prospective study [24], they showed that FLI was associated with cardiovascular disease-related mortality and cancer-related mortality after 15-year follow-up of 2,074 Caucasian individuals in three Italian municipalities. Another study of Gastaldelli et al [25] found that increased intima media thickness, coronary heart disease risk (evaluated by Framingham risk score), and reduced insulin sensitivity were associated with high values of FLI in RISC population (also a European Caucasian population). According to International Classification of Diseases [26], total cardiovascular diseases included not only CAD but also other diseases such as disorders of the peripheral vascular system, disease of the aorta and its branches, and congenital heart disease, etc (ICD-10 codes I00–I99, Q20– Q28). CAD is limited as the blockage of one or more of the coronary arteries that supply blood to the heart which usually is caused by atherosclerosis. However, no study has evaluated the relationship between FLI and CAD till now. In order to differentiate CAD with other cardiovascular disorders, we chose coronary angiography as a golden standard. Unfortunately, we could not find the correlation between FLI and CAD in either gender. Because there was no information of coronary angiography provided in the other two studies, the certain relationship between FLI and CAD in those studies could not be speculated. The patients enrolled in our study were suspicious of CAD, but not the general population. This may be one of the reasons leading to the differences in results between our study and the aforementioned two studies [24,25]. On the other hand, patients in the control group (non-CAD patients) with negative findings of coronary angiography were different from subjects in general population; some of them might have other cardiovascular diseases, which might lead to the underestimation of the relationship between FLI and CAD.

This cross-sectional study had some limitations. First, the genders in our study could not be balanced in CAD group: ratio of male to females was about 3:1. This is due to the fact that CAD is more prevalent in males. Second, CAD usually progresses for many years starting from metabolic changes which may simultaneously affect the occurrence of fatty liver. Actually, these two diseases do not progress in parallel. In this study, the patients were all diagnosed as CAD at first-time by coronary artery angiography. However, the exact duration of the disease could not be estimated. Third, majority of the patients in the study were prescribed with anti-hyperlipidemia drugs such as statin, etc. The potential effect of these drugs on both CAD and fatty liver disease cannot be excluded. Therefore, we performed the analysis adjusted for both anti-hyperlipidemia and diabetic mellitus to minimize such possibility. Finally, Neyman bias may exist to affect the results due to the nature of study design.

## Conclusions

FLI showed good correlation with NAFLD in patients who underwent coronary angiography. Although we did not find a relationship between FLI with newly diagnosed CAD in the present study, our results did not exclude the presence of correlation between FLI and cardiovascular disease which was already proved in general population [24,25]. With the rapid change in lifestyle, CAD becomes healthy and economic burden and one of the leading causes of death in Chinese. Recently, a cross-sectional study representative sample of 46,239 Chinese adults over 20 years of age showed that the awareness, treatment, and control of high total cholesterol were 11.0%, 5.1%, and 2.8%, respectively [27]. This indicates that a large sub-population would be susceptible to the development of CAD. The certain relationship between FLI and CAD is still awaited to be further clarified by the results of some large, prospective studies in Chinese population in the future.

## Competing interests

The authors declare that they have no competing interests.

## Authors’ contributions

ZYJ and LFY designed the study and wrote the manuscript. ZYJ, CYX and LFY analyzed the data. CYX, XXC, WWL, LYS and XBY collected the data. All authors were involved in editing the manuscript. All authors read and approved the final manuscript.

## Pre-publication history

The pre-publication history for this paper can be accessed here:

http://www.biomedcentral.com/1471-230X/13/110/prepub

## Supplementary Material

Additional file 1 **Fatty liver index correlates with non-alcoholic fatty liver disease, but not with newly diagnosed coronary artery atherosclerotic disease in Chinese patients. Table S1** Clinical characteristics between NAFLD group and non-NAFLD group. **Table S2** Clinical characteristics among different FLI categories*. **Table S3** Clinical characteristics between CAD group and non-CAD group. Click here for file
