# Supplementary material for: Fatty liver index correlates with non-alcoholic fatty liver disease, but not with newly diagnosed coronary artery atherosclerotic disease in Chinese patients
Source: BMC Gastroenterol. 2013 Jul 8;13:110. doi: 10.1186/1471-230X-13-110 (PMC3710104; doi:10.1186/1471-230X-13-110)
Supplement: Additional file 1 — Fatty liver index correlates with non-alcoholic fatty liver disease, but not with newly diagnosed coronary artery atherosclerotic disease in Chinese patients. Table S1 Clinical characteristics between NAFLD group and non-NAFLD group. Table S2 Clinical characteristics among different FLI categories*. Table S3 Clinical characteristics between CAD group and non-CAD group. [file 1471-230X-13-110-S1.doc]

**Supplementary Tables**

**Fatty liver index correlates with non-alcoholic fatty liver disease, but not with newly diagnosed coronary artery atherosclerotic disease in Chinese patients**

Zhao-Yan Jiang^1‡^, Chen-Ying Xu^2‡^, Xian-Xing Chang^2^, Wei-Wei Li^2^, Lu-Ying Sun^2^, Xiao-Bo Yang^2^, Li-Fen Yu^2§^

**Table S1 Clinical characteristics between NAFLD group and non-NAFLD group**

|  | Non-NAFLD | NAFLD |
| --- | --- | --- |
| N (male/female) | 365(193/172) | 209(113/96) |
| Age(year) | 65.3±0.6 | 62.9±0.8* |
| BMI(kg/m^2^) | 23.3±0.2 | 25.9±0.2** |
| WC(cm) | 82.6±0.5 | 88.0±0.6** |
| ALT(IU/L) | 24.9±1.2 | 31.4±1.8** |
| AST(IU/L) | 29.8±2.2 | 31.6±2.7 |
| AKP(IU/L) | 63.9±1.0 | 61.9±1.3 |
| GGT(IU/L) | 28.2±1.6 | 34.6±2.0** |
| TC(mmol/L) | 3.75±0.08 | 3.90±0.10 |
| TG(mmol/L) | 2.15±0.08 | 2.89±0.27** |
| HDL(mmol/L) | 1.27±0.03 | 1.12±0.03** |
| LDL(mmol/L) | 2.61±0.04 | 2.58±0.06 |
| FPG(mmol/L) | 5.18±0.07 | 5.54±0.11** |
| Hypertension (n, %) | 237(64.9%) | 161(77.0%)** |
| Diabetic mellitus (n, %) | 60(16.4%) | 53(25.4%)** |
| Dyslipidemia (n, %) | 102(27.9%) | 87(41.6%)** |
| Anti-hyperlipidemia drugs (n, %) | 318(87.1%) | 192(91.9%) |
| Anti-diabetic mellitus drugs (n %) | 39(10.7%) | 39(18.7%)** |

* *P* < 0.05 and ** *P* < 0.01 between two groups. Continuous data were expressed as means± S.E.M.

**Table S2 Clinical characteristics among** different FLI categories*

|  | FLI≤30 | FLI 30-60 | FLI≥60 |
| --- | --- | --- | --- |
| N (male/female) | 483(211/272) | 145(103/42) | 85(71/14) |
| Age(year) | 66.3±0.5^a^ | 61.2±0.9b^c^ | 59.2±1.1^c^ |
| BMI(kg/m^2^) | 23.0±0.1^a^ | 26.3±0.2^b^ | 28.7±0.3^c^ |
| WC(cm) | 82.1±0.4^a^ | 89.2±0.6^b^ | 95.6±0.9^c^ |
| ALT(IU/L) | 25.0±0.9^a^ | 29.7±1.9^bc^ | 34.5±3.7^c^ |
| AST(IU/L) | 29.7±1.7 | 32.5±3.2 | 32.4±5.7 |
| AKP(IU/L) | 62.7±0.9^a^ | 65.0±1.7 | 68.4±2.1^b^ |
| GGT(IU/L) | 27.3±1.1^a^ | 38.9±4.4^bc^ | 37.0±3.0^c^ |
| TC(mmol/L) | 3.99±0.07^a^ | 3.54±0.13^b^ | 4.00±0.15^a^ |
| TG(mmol/L) | 2.10±0.12^a^ | 2.75±0.14^bc^ | 3.03±0.18^c^ |
| HDL(mmol/L) | 1.27±0.02^a^ | 1.08±0.02^bc^ | 1.02±0.03^c^ |
| LDL(mmol/L) | 2.62±0.04 | 2.58±0.07 | 2.73±0.10 |
| FPG(mmol/L) | 5.32±0.07 | 5.38±0.10 | 5.68±0.23 |
| FLI | 10.3±0.4^a^ | 42.7±0.7^b^ | 77.9±1.2^c^ |
| Hypertension (n, %) | 328(67.9%) | 106(73.1%) | 60(70.6%) |
| Diabetic mellitus (n, %) ** | 142(29.4%) | 63(43.4%) | 39(45.9%) |
| Dyslipidemia (n, %) | 97(20.1%) | 32(22.1%) | 10(11.8%) |
| Anti-hyperlipidemia drugs (n, %) | 427(88.6%) | 128(88.3%) | 78(91.8%) |
| Anti-diabetic mellitus drugs(n, %) | 69(14.3%) | 18(12.4%) | 6(7.1%) |

* Different letters indicated differences between two groups by post-hoc LSD analysis, *P* < 0.05. ** *P* < 0.05 by *x*^2^ test. Continuous data were expressed as means± S.E.M.

**Table S3 Clinical characteristics between CAD group and non-CAD** group

|  | Non-CAD | CAD |
| --- | --- | --- |
| N (male/female) | 482 (215/267) | 231(171/60) |
| Age(year) | 62.8±0.5 | 67.9±0.7** |
| BMI(kg/m^2^) | 24.4±0.1 | 24.3±0.2 |
| WC(cm) | 84.1±0.4 | 87.3±0.7** |
| ALT(IU/L) | 26.1±1.1 | 29.1±1.4 |
| AST(IU/L) | 24.5±0.7 | 43.2±4.3** |
| AKP(IU/L) | 64.1±0.9 | 63.4±1.3 |
| GGT(IU/L) | 30.3±1.6 | 31.8±1.5 |
| TC(mmol/L) | 3.69±0.07 | 4.34±0.07** |
| TG(mmol/L) | 2.62±0.13 | 1.77±0.06** |
| HDL(mmol/L) | 1.24±0.02 | 1.10±0.02 |
| LDL(mmol/L) | 2.64±0.04 | 2.60±0.06 |
| FPG(mmol/L) | 5.22±0.05 | 5.70±0.13** |
| FLI | 24.7±1.1 | 25.3±1.7 |
| Hypertension (n, %) | 328(68.0%) | 166(71.9%) |
| Diabetic mellitus (n, %) | 94(20.8%) | 45(19.5%) |
| Dyslipidemia (n, %) | 156(32.3%) | 88(38.1%) |
| Anti-hyperlipidemia drugs (n, %) | 412(85.5%) | 221(95.7%)* |
| Anti-diabetic mellitus drugs (n, %) | 62(12.9%) | 31(13.4%) |

** *P*< 0.01, CAD group compared with non-CAD group, by t-test. Continuous data were expressed as means ± S.E.M.
